# SATB1 Expression Is Associated with Biologic Behavior in Colorectal Carcinoma In Vitro and In Vivo

**DOI:** 10.1371/journal.pone.0047902

**Published:** 2013-01-11

**Authors:** Jie Zhang, Baogang Zhang, Xumei Zhang, Yingui Sun, Xiaolong Wei, Michael A. McNutt, Shijun Lu, Yuqing Liu, Donghong Zhang, Mingyu Wang, Zhijuan Lin, Na Niu

**Affiliations:** 1 Department of Human Anatomy, Weifang Medical University, Weifang, China; 2 Department of Pathology, Weifang Medical University, Weifang, China; 3 Department of Pathology, affiliated hospital of Weifang Medical University, Weifang, China; 4 Department of Anesthesia, Weifang Medical University, Weifang, China; 5 Department of Pathology, Cancer hospital of Shantou University Medical College, Shantou, China; 6 Department of Pathology, School of Basic Medical Sciences, Peking University, Beijing, China; 7 Department of Clinical Laboratory, Peking Union Medical College Hospital and Peking Union Medical College, Beijing, China; 8 Department of Neurology, Weifang Medical University, Weifang, China; 9 Key Laboratory for Immunology in Universities of Shandong Province, Weifang Medical University, Weifang, China; University of Porto, Portugal

## Abstract

There is increasing evidence that Special AT-rich sequence-binding protein 1 (SATB1) is aberrantly expressed in several cancers and is correlated with clinicopathologic parameters in these tumors. In this study, we showed over-expression of SATB1 in 80 cases of colorectal cancer and in 3 colorectal cancer cell lines and found expression levels were strongly associated with tumor differentiation and stage. Expression levels of SATB1 protein were higher in poorly-differentiated as compared with well-differentiated cell lines, and both quantity and distribution patterns of SATB1 were associated with tumor differentiation and pTNM stage. Strikingly, we further investigated the effect of down regulation of SATB1 expression on malignant phenotypic features in colorectal cancer cells in vitro, and showed that SABT1 down-regulation negatively affected growth potential, anchorage-independent colony formation and cancer cell invasion, and resulted in increased apoptosis. SATB1 expression was positively associated with the expression of various biological and genetic markers, including Cyclin D1, MMP-2, NF-κB, and PCNA, and was associated with loss of APC and BRAF^V600E^. These findings suggest that SATB1 is involved in the carcinogenesis, development and progression of colorectal cancer.

## Introduction

Special AT-rich sequence-binding protein 1 (SATB1) which was first identified in T cells is important in chromatin organization, and is involved in the regulation of hundreds of genes [1], [2]. Under physiological conditions, it plays major roles in T-cell development, early erythroid differentiation, cellular homeostasis and response to various stimuli [3]–[6]. Recently, SATB1 has been found to be overexpressed in several kinds of malignant tumors, such as breast carcinoma [7], gastric carcinoma, laryngeal, hepatocellular, ovarian and bladder carcinoma and in cutaneous malignant melanoma [8]–[14], and evidence suggests SATB1 overexpression is significantly associated with clinicopathologic findings in these neoplasms [3]. In breast carcinoma, SATB1 regulates the expression of more than 1000 genes which are involved in 61 kinds of biological activity such as proliferation, apoptosis, DNA organization, electron transport, protein generation, receptor activity and so on. It has also been demonstrated to be a key gene in metastasis as well as an independent prognostic factor [7].

Colorectal carcinoma (CRC) is the second leading cause of cancer death in adults worldwide [15]. The molecular mechanisms of CRC have been extensively studied and there have been advances in diagnostic methodology and therapy in recent decades, however the overall survival of patients with higher stage CRC has not improved significantly. Stage IV CRC is typically incurable and death most commonly results from distant metastases in these patients [16]. Current knowledge of the specific mechanisms of CRC metastasis is limited, however the function of SATB1 in progression and metastasis of breast cancer raises the possibility it may function in a similar manner in CRC.

SATB1 in carcinoma of the rectum has been shown to be associated with depth of invasion, TNM stage and differentiation in a previous study [14]. The present study confirms these findings in carcinomas of both the colon and the rectum, and more importantly analyzes the correlation of phenotypic and genotypic features of CRC related to SATB1 expression. We further demonstrate SABT1 expression significantly affects malignant phenotype features of CRC in vitro, including tumor growth potential, anchorage-independent colony formation, cancer cell invasion, and apoptosis. We also show SATB1 cellular localization is correlated with tumor differentiation. This molecule may be useful as a marker for progression and metastasis in CRC.

## Materials and Methods

### Tissue Samples

Fresh and paraffin-embedded tissues from 80 cases of sporadic CRC tumor resection with paired normal colorectal mucosa specimens were collected at the Affiliated Hospital of Weifang Medical University. Cancer differentiation for the purpose of this study was graded as either well to moderately differentiated or poorly differentiated. This study was approved by the ethical committee of Weifang Medical University and written consent for participation in the study was obtained from all patients.

### Cell Lines

Human CRC cell lines of differing tumor grade (HT29, well differentiated; SW480, moderately differentiated; LOVO, poorly differentiated) [17] and Jurkat cells were purchased from American Type Culture Collection (ATCC). Jurkat cells which are a T lymphocytic leukemia cell line were used as a positive control for SATB1 expression.

### Immunohistochemistry

To evaluate expression of SATB1 and other cancer-related proteins, immunohistochemistry (IHC) was performed on 4 micron tissue sections of CRC and paired normal tissues as described previously [18], [19]. Information about the primary antibodies used in this study including CEA (marker for CRC cell), SATB1, P53, PCNA, Bcl-2, MMP-2, NF-κB, Cyclin D1 and APC is given in Table S1. Horseradish peroxidase (HRP)-conjugated anti-mouse/rabbit IgG (PV9000 immunohistochemistry Kit, Zymed Laboratories, San Francisco, CA, USA) was used as the secondary antibody. T lymphocytes in the colorectal wall served as a positive control for SATB1. Normal goat serum was substituted for primary antibody as a negative control, and normal colon tissues were also used as negative controls. 3-amino-9-ethyl-carbazole (AEC) or Nitro blue tetrazolium chloride/5-Bromo-4- chloro-3-indolyl phosphate (NBT/BCIP) was used to visualize positive reactions.

### Scoring Immunoreactivity

Stained sections were microscopically examined and scored independently by 3 of the authors (N.N, W.Y, and Z.C) without knowledge of the biological and clinicopathologic data. Nuclear proteins (SATB1, PCNA, NF-κB, Cyclin D1) were scored according to the percentage of positive nuclei [20]. Less than 25% positive was scored as “negative”, and ≥25% was scored as “positive” (scoring adopted from a previously used scored system) [20]. Cytoplasmic proteins (P53, Bcl-2, MMP-2, APC) were given an overall staining score by taking the sum of the score of percentage of positive cells (scored as 0 = <5%, 1 = 5–25%, 2 = 25–50%, 3 = >50%) and staining intensity (scored as 0, absence of signal; 1, light red or pink; 2, red; 3, dark red).

### Immunofluorescence and Confocal Microscopy

To investigate the expression and distribution of SATB1 in colorectal cancer lines, double-label immunofluorescence (IF) with antibodies to SATB1 and CEA was performed as previously described [18], [21]. Monoclonal mouse anti-human SATB1 (1∶100, BD Biosciences, San Jose, CA, USA) and polyclonal rabbit anti-human CEA (Zymed Laboratories, San Francisco, CA, USA) were used as primary antibodies. PBS instead of primary antibody was used as a negative control. Goat anti-mouse IgG-TRITC (1∶100, Jackson, West Grove, PA, USA) and goat anti-rabbit IgG-FITC (1∶100, Jackson) were employed as secondary antibodies. Nuclei were stained with Hoechst 33342 (1∶500; Sigma). After mounting with glycerol/PBS (9∶1), slides were evaluated with a FV1000 confocal microscopy (Olympus, Tokyo, Japan).

### cRNA Probe Preparation and In Situ Hybridization

A specific cRNA antisense probe directed against human SATB1 was prepared and in situ hybridization (ISH) was performed as described previously [7], [21]. T lymphocytes in the colorectal wall were used as a positive control, and slides were incubated with sense probes for negative controls.

### Real Time RT-PCR

Total RNA was isolated from cell lines (Jurket, HT29, SW480, LOVO) using TRIzol reagent (Invitrogen, Carlsbad, CA, USA) according to the manufacturer’s protocol. RQ1 RNase-free DNase (Promega, Madison, WI, USA) was used to treat RNA samples to exclude contamination by genomic DNA. Five micrograms of total RNA extract were reverse transcribed with oligo (dT)_18_ primer (Fermentas, Burlington, Ontario, Canada) using SuperScript III Reverse Transcriptase (Invitrogen) according to the manufacturer’s protocol.

To check the levels of SATB1 transcripts in CRC cell lines, relative quantification was performed in triplicate with real-time PCR (qPCR) using SYBR Premix Ex Taq (TaKaRa, Dalian, China) containing SYBR Green I, TaKaRa Ex Taq, dNTP mixture and Mg^2+^. Primers used for SATB1 amplification/quantification were identical to those described previously [7]. β-actin was used for normalization. Blank samples in which the template was omitted were used controls. Real-time PCR was performed with the ABI PRISM 7500 Real-Time PCR system (Applied Biosystems, Foster City, CA, USA), using the following protocol: denaturation (95°C for 10 s); amplification and quantification cycles repeated 35 times (95°C for 5 s, 52°C for 20 s, 72°C for 34 s with a single fluorescence measurement) and a dissociation stage.

### Genomic DNA Extraction and PCR

B-raf gene mutation (Exon 15, BRAF^V600E^) is the key event of the serrated pathway in carcinogenesis of sporatic CRC [22]. The status of the B-raf gene was evaluated in the current study. Genomic DNA was extracted and purified from fresh CRC tissue with the Wizard™ SV Genomic DNA Purification System (Promega, Madison, WI, USA) according to the manufacturer’s protocol. PCR was carried out with LA Taq polymerase (TaKaRa, Dalian, China). Specific primers for B-raf exon 15 were as follows: TCATAATGCTTGCTCTGATAGGA (sense), GGCCAAAAATTTAATCA- GTGGA (antisense). One microgram of genomic DNA was used as the template and the PCR amplification began with incubation at 95°C for 5 min, proceeded for 35 cycles at 95°C for 1 min, then 52°C for 30 s, and 72°C for 30 s. The final cycle was followed by an extension of 10 min at 72°C.

### SSCP Analysis

PCR products of the B-raf gene were analyzed for mutations in exon 15 with the MDE^®^ Heteroduplex Kit (Lonza, Rockland, ME, USA). In brief, 1 µl of PCR product was added to 9 µl stop solution (Lonza), heated at 94°C, then kept on ice until it loaded onto the MDE gel (Lonza). Samples were then run through the gel at 6 W for 14 hours at 4°C and the gel was stained with silver. Abnormal bands were observed.

### Knockdown of SATB1 Expression

Knockdown vectors (siRNA-SATB1) and a corresponding control (siRNA-Control) were gifts from Dr. Yujie Sun (Key Laboratory of Functional Genomics of Jiangsu Province, Nanjing Medical University). Vectors were transfected into LOVO cells using Lipo2000 (Invitrogen) and stable clones were selected by incubation with 1 mg/ml G418.

### Western Blot

Nuclear protein of stably transfected LOVO cell lines (40 µg/lane) was analyzed with 10% SDS-PAGE. Mouse anti-human SATB1 (1∶1000, BD Biosciences) were used as the primary antibody.

### Cell Proliferation and Apoptosis Analysis

The MTS cell proliferation assay (CellTiter 96 AQ One Solution Cell Proliferation Assay Kit, Promega) was carried out in a 96-well plate according to the manufacturer’s instructions. LOVO cells in which SATB1 was knocked down were incubated for one week and the OD490 was recorded and analyzed at 24 hour intervals [23]. Empty vectors were used as a negative control. Additional negative controls included treatment with no vector and a blank control where no cells were seeded. All experiments were performed in triplicate.

For analysis of apoptosis, LOVO cells with SATB1 knock down were analyzed with the Annexin V-PI kit (Peking University Human Disease Genomics Research Center, Beijing, China) as described previously [24]. Empty vectors were used as a negative control.

### Soft Agar Assay on Clone Formation

The soft agar assay was carried out as described previously [25]. Empty vectors were used as a negative control.

### Cell Invasion Assay

A modified cell migration assay was performed with LOVO cells as described previously [26]. The membrane was stained with H&E. Five random fields per membrane were photographed with a BX51 microscope (Olympus) at ×400 magnification. The invading cells were counted and average numbers were calculated for each membrane.

### Statistical Analysis

Correlation between SATB1 expression and CRC-relative biological variables, differences in SATB1 expression in normal mucosa samples and primary tumors, and differences in SATB1 expression among various clinicopathologic features were tested using the χ^2^ test with SPSS 12.0 software. Two-sided p values of less than 5% were considered statistically significant.

## Results

### Expression and Distribution of SATB1 Protein and mRNA in Human CRC Tissues and Cell Lines

**Figure 1:**
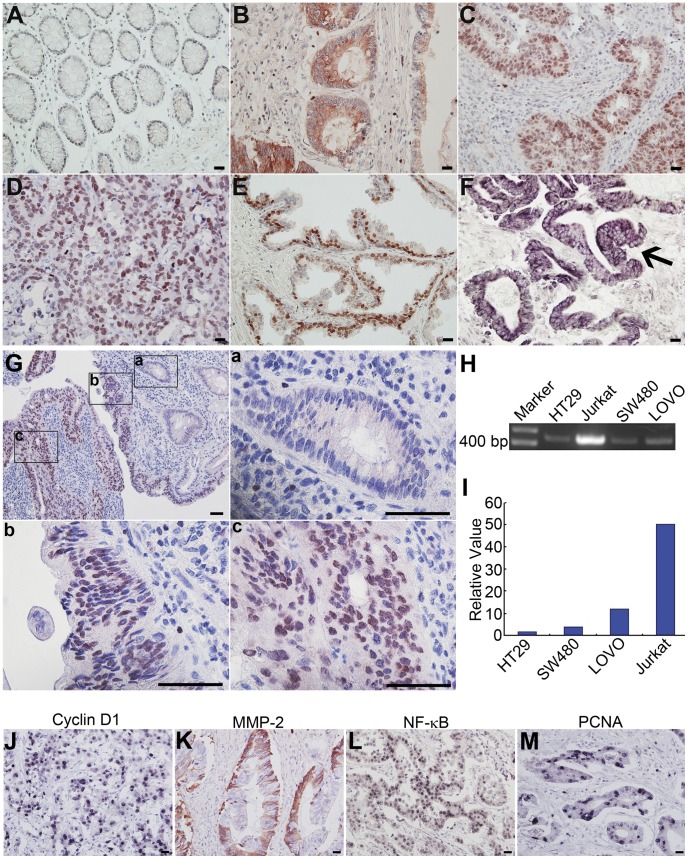
SATB1 expression in CRC tissues and cell lines. **A–E:** SATB1 protein expression (positive signals show red staining with AEC) in matched normal colorectal epithelium (**A**); and well (**B**), moderately (**C**) and poorly differentiated (**D, E**) CRC tissues. In normal colon tissue (**A**) positive staining is seldom found in the normal glandular epithelium. T lymphocytes in the lamina propria are positive. In CRC tissues (**B–E**) positive signals for SATB1 are distributed in the cytoplasm or in nuclei or both in carcinoma epithelial cells. **F:** Expression of SATB1 mRNA in cytoplasm of CRC cells (arrows, purple signals stained with NBT/BCIP). **G:** SATB1 protein expression in one sample of CRC tissue and in the peri-tumoral tissue (red signals stained with AEC). **a, b** and **c** are higher magnifications of the corresponding regions in the upper left panel. SATB1 is negative in the peri-tumoral normal glandular epithelium (**a**). More cancer cells are positive for SATB1 in the central zone of the neoplasm (**c**) than in the periphery (**b**) of the cancer and normal tissue. **H:** Agarose gel electrophoresis of RT-PCR amplification products following mRNA extraction from three cell lines and a positive control. **I:** Relative levels of SATB1 in CRC cell lines using qRT-PCR. **J–M:** Cyclin D1(**J**), MMP-2(**K**), NF-κB (**L**) and PCNA(**M**) expression in CRC tissues (**J, L, M:** purple signals stained with NBT/BCIP; **K:** red signals stained with AEC). Bars = 50 µm.

In 80 cases of cancer, SATB1 protein was positive in 47 (58.75%), which was significantly higher than in the matched normal colon mucosa (2.5%, P<0.01). In the matched normal mucosa, positive signals for SATB1were found only in T lymphocytes and very rarely in epithelial cells (Figure 1A). In cancer tissue, distribution of SATB1 showed differences associated with tumor differentiation. In well differentiated adenocarcinomas, positive signal was found mainly in the cytoplasm of epithelial cells, and infrequently in nuclei (Figure 1B). In moderately differentiated carcinomas, positive signals localized mainly in the nucleus with some weak cytoplasmic staining (Figure 1C). Poorly differentiated tumors showed positive signal only in the nuclei of cancer cells (Figure 1D and E). Positive signals for SATB1 mRNA localized as expected in the cytoplasm of cancer cells (Figure 1F). Characteristic SABT1 distribution was illustrated in Figure 1G where SATB1 protein was negative or weakly positive in the cytoplasm of non-neoplastic epithelium at the tumor periphery (Figure 1G a), while the cancer cells show nuclear positivity (Figure 1G b, c). The frequency of SATB1 positive cells was also lower at the periphery of cancer tissue (Figure 1G b) than those in the central zones of the cancer tissue (Figure 1Gc).

Using confocal microscopy, SATB1 protein was found localized mainly in the nucleus and a small amount was also distributed in the perinuclear cytoplasm as shown in Figure S1). SATB1 transcripts were identified in all three CRC cell lines via RT-PCR as shown in Figure 1 H, and the intensity of the positive bands was less than that seen in Jurkat cells bands. qPCR was used to quantify SATB1 transcripts in these four cell lines, and SATB1 was highly expressed in LOVO, moderately expressed in SW480, and showed low expression in HT29 corresponding to the relative differentiation of these tumor cells (poorly differentiated, moderately and well differentiated respectively) (Figure 1I).

### Correlation of SATB1 Expression with Clinicopathologic Features, Biologic Markers and BRAF Mutation in CRC

SATB1 expression showed correlation with tumor grade, depth of invasion and overall TNM stage in primary tumor cells as shown in Table 1. Frequency of SATB1 expression was higher in poorly differentiated adenocarcinoma (84.6%) than in well and moderately differentiated neoplasms (51.9%, P = 0.035). SATB1 expression also showed significant differences with depth of tumor invasion (T1+T2 vs. T3+T4, 48.4% vs. 65.3%, P = 0.024) and with pTNM stage (stage I+II vs. III + IV stages (62.5% vs. 77.1%, P = 0.047). SATB1 expression showed no significant correlation with patient age, gender, tumor location (colon vs. rectum) or growth pattern (expansile vs. infiltrative) (all P>0.05).

**Table 1:** Correlations Between SATB1 Expression and Clinicopathological Features of CRC.

Variable	n	SATB1 expression		P value*
		Negative	Positive	
Age, years				0.491
<40	7	3 (42.9)	4 (57.1)	
40–60	51	22 (43.1)	29 (56.9)	
>60	22	8 (36.4)	14 (63.6)	
Gender				0.717
Male	43	17 (39.5)	26 (60.5)	
Female	37	16 (43.2)	21 (56.8)	
Tumor location				0.755
Colon	35	14 (40.0)	21 (60.0)	
Rectum	45	19 (42.2)	26 (57.8)	
Growth pattern				0.182
Expansive	17	8 (47.1)	9 (52.9)	
Infiltrative	63	25 (39.7)	38 (60.3)	
Differentiation				0.035
Better	54	27 (50.0)	27 (50.0)	
Worse	26	6 (26.9)	20 (73.1)	
TNM Stage				0.047
I+II	32	12 (37.5)	20 (62.5)	
III+V	48	11 (22.9)	37 (77.1)	
Depth of Invasion				
T1+T2	31	16 (51.6)	15 (48.4)	0.024
T3+T4	49	17 (34.7)	32 (65.3)	

Figures in parentheses are percentages.

* χ^2^ test.

Table 2 presents the relationship between SATB1 expression and these molecules found in this investigation. SATB1 expression showed positive correlation with expression of Cyclin D1 (P = 0.045) (Figure 1J), MMP-2 (P = 0.039) (Figure 1K), NF-κB (P = 0.05) (Figure 1L), and PCNA (P = 0.038) (Figure 1M), and was showed negative correlation with APC expression (P = 0.041) (Figure S2 A and B). There was no significant correlation of SATB1 with expression of Bcl-2 or p53 (both P>0.05). BRAF^V600E^ was evaluated in 80 cases of CRC with SSCP analysis. As shown in Figure S2 C and Table 2, a positive BRAF^V600E^ ration was found in a total 13.75% of cases (11/80). SATB1 overexpression was significantly correlated with BRAF^V600E^ (P = 0.016).

**Table 2:** Relationships Between SATB1 Expression and Various Biological and Genetic Factors of CRC.

Variable	n	SATB1 expression		P value*
		Negative	Positive	
Bcl-2				
Negative	46	20 (43.5)	26 (56.5)	0.061
Positive	34	13 (38.2)	21 (61.8)	
Cycline D1				
Negative	31	10 (32.3)	21 (67.7)	0.045
Positive	49	23 (46.9)	26 (53.1)	
MMP-2				
Negative	57	26 (45.6)	31 (54.4)	0.039
Positive	23	7 (30.4)	16 (69.6)	
NF-κB				
Negative	54	24 (44.4)	30 (55.6)	0.05
Positive	26	9 (34.6)	17 (65.4)	
P53				
Negative	59	22 (37.3)	37 (62.7)	0.131
Positive	21	11 (52.4)	10 (47.6)	
Ki 67				
Negative	40	14 (35.9)	26 (64.1)	0.038
Positive	40	19 (30.0)	21 (70.0)	
APC				
Negative	52	19 (36.5)	33 (63.5)	0.041
Positive	28	14 (50.0)	14 (50.0)	
BRAF^V600E^				
Negative	69	31 (44.9)	38 (55.1)	0.016
Positive	11	2 (18.2)	9 (81.8)	

Figures in parentheses are percentages.

* χ^2^ test.

### SATB1 Augments Proliferation and Invasive Capacity in CRC Cells In Vitro

**Figure 2:**
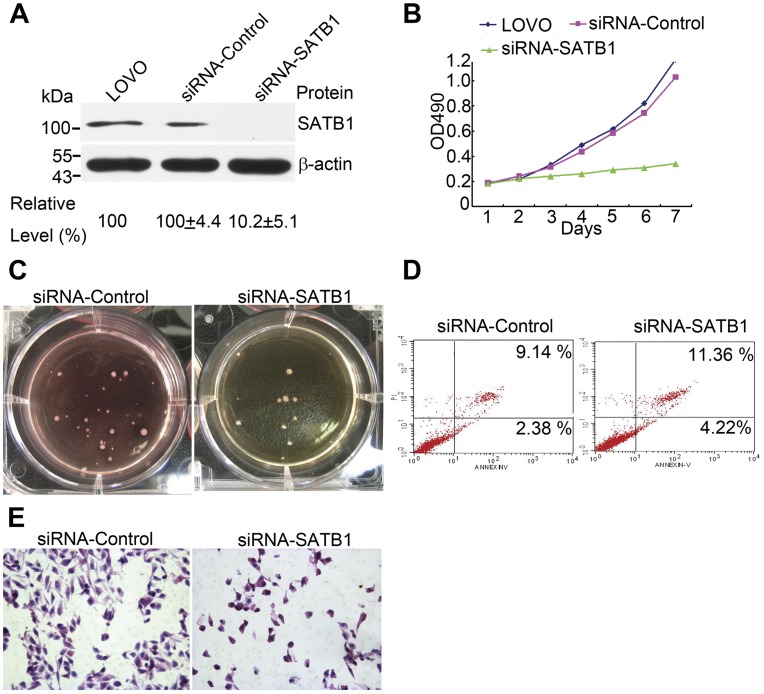
Reduction of SATB1 expression by siRNA affects the biologic behavior of CRC cells. **A:** Effect of SATB1 down-regulation. **B:** Evalaution of cell proliferation with the MTS essay shows that SATB1 down-regulation significantly inhibits growth of LOVO cells. **C:** Results of soft agar colony formation essay in LOVO cells with SATB1 down regulation. **D:** AnnexinV-PI analysis shows that the percentage of apoptotic LOVO cells is higher after SATB1 down-regulation as compared with the empty vector control. **E:** SATB1 down-regulation suppresses the invasion potential of CRC cells. Bars = 50 µm.

The effects of SATB1 down-regulation with siRNA are shown in Figure 2A. In LOVO cells the relative protein levels of SATB1 in untreated cells, cells with siRNA-control and cells with siRNA-SATB1 were 100, 100±4.4, and 10±5.1 respectively, indicating that there was an effective decrease of about 90% in SATB1 protein with the siRNA-SATB1. Using the MTS essay, the rate of proliferation in LOVO cells with down regulated SATB1 decreased markedly. As shown in Figure 2B, the difference in proliferative rate in the control and test cells became apparent on the third day of subculture and this difference continued to increase throughout seven days of subculture. The soft-agar colony-formation essay was carried out to investigate the effects of SATB1 on anchorage independent growth in CRC cells. The average number of colonies formed in SATB1 down-regulated LOVO cells and the untreated LOVO control cells were 10.7±3.2 and 30.2±4.6 (P<0.05, Figure 2C).

We also studied the effect of SATB1 on apoptosis in colorectal cancer cells using flow cytometry with the Annexin V-PI kit. The results (Figure 2D) show that SATB1 down-regulation resulted in a higher rate of apoptosis in LOVO cells (14.10±3.46) % as compared with the control (8.62±4.13)% (P<0.05). This suggests SATB1 expression in colorectal promotes tumor survival.

Additionally, to determine whether SATB1 is associated with capacity for invasion in colorectal cancer cells, a cell invasion assay using matrigel and a multiporous membrane was carried out with LOVO cells in which SATB1 was down regulated. As shown in Figure 2E, the average number of invading cells was significantly lower in the SATB1 down-regulated cells (34.5±4.7), than in the untreated LOVO control cells (119.1±3.9, P<0.05). These results suggest that SATB1 supports the capacity for invasion in colorectal cancer cells.

## Discussion

As a tissue-specific MAR-binding protein, SATB1 functions by fixing chromatin loop to the nuclear matrix via recognition of and binding with DNA matrix attachment regions (MARs). It then recruits transcription factors, and reorganizes and remodels chromatin [3]. As a global chromatin organizer and transcription factor, SATB1 is a key gene in T cell development [27] and erythoid differentiation [5].

Over-expression of SATB1in human breast cancer gives rise to up-regulation of 456 genes, and down-regulation of 409 genes and these genes are involved in more than 50 biological activities such as apoptosis, cell-cell adhesion, carbohydrate metabolism, cell differentiation, proliferation, DNA recombination and repair, immune response, kinase activity, oxidoreductase activity and signal transduction. SATB1 is involved in tumor progression and has been suggested to be an independent prognostic factor [7]. In its role as an important protein in chromatin reorganization, SATB1 can up-regulate expression of PARP1 which takes part in single-stranded DNA repair resulting in synthetic lethality in BRCA1/2 defective cancers.PARP1 inhibitors have been successfully applied in clinical treatment for breast and ovarian cancers [28], [29]. Although BRCA1/2 mutations can incite genomic instability and are strong predictors of breast cancer [30]–[32], SATB1 abnormality is unrelated to BRCA1/2 mutations [7] indicating that SATB1 affects DNA repair in a manner which is independent of BRCA1/2. SATB1 has subsequently been found to be aberrantly expressed in several malignant tumors, where its expression is associated with various clinicopathologic factors. These results suggest that SATB1 may be useful as a new marker for tumor progression and prognosis.

In the present study, we confirmed SATB1 overexpression is significantly correlated with tumor differentiation, depth of invasive and pTNM stage in CRC, consistent with the findings of the previous study by Meng et al [14].

CRC is a complex neoplasm which results from several factors such as genetic alteration, chromosomal instability, and activity of growth factor pathways [33]. Three genomic abnormalities are crucial in CRC tumourigenesis, including chromosomal instability (CIN, affects proto-oncogenes and tumor suppressor genes), epigenetic instability (resulting from hypermethylation-induced silencing of tumor suppressor genes) and microsatellite instability (MSI) [34]. Raf/MEK/ERK (MAPK) signal transduction is an important mediator of these genetic and epigenetic changes. BRAF^V600E^ is thought to be pivotal in CpG methlylation, and in MSI and DNA mismatch repair [35]–[37], which are initial events in CRC carcinogenesis. In the present study, we evaluated BRAF^V600E^ and found it was significantly correlated with SATB1 over-expression. As a key protein in DNA remodeling, SATB1 may be involved in genetic mutations such as BRAF^V600E^.

We also studied the association of SATB1 expression with various molecules which have been previously identified as biological and prognostic markers in colorectal carcinoma, including Bcl-2 (anti-apoptotic protein), Cyclin D1 (proliferation marker), MMP-2 (invasion marker), NF-κB (anti-apoptotic factor and nuclear transcription factor), P53 (tumor suppression gene), APC (tumor suppression gene) and PCNA (proliferation marker). In several studies over-expression (Cyclin D1, NF-κB, PCNA, MMP-2) or loss of expression (Bcl-2, APC) of these molecules have been found to be associated with more aggressive tumor growth in colorectal cancer [17], [38]–[40]. Bcl-2, APC, P53 are also involved in DNA repair [40]–[42]. In the present study, SATB1 expression showed positive correlation with expression of Cyclin D1, MMP-2, NF-κB, and PCNA and APC loss. As these proteins are important prognostic factors in colorectal cancer [39], this correlation with SATB1 expression additionally supports the concept SATB1 is a driver of the malignant phenotype in colorectal carcinoma.

Our in vitro findings with one CRC cell line were consistent with this strong association of stage, invasion and differentiation with SATB1 expression. We evaluated the effect of decreased expression of SATB1 via siRNA gene silencing on the biologic behavior of high grade CRC cells. Our results showed that SATB1 augments proliferation, soft agar colony formation and capacity for invasion, as well as decreased apoptosis in these high grade cells. These results strongly support the view that over expression of SATB1 plays a significant important role in CRC progression and metastasis.

In addition we found that IHC signals for SATB1 protein become stronger and their distribution changes from cytoplasmic to nuclear in poorly differentiated CRC tumors as compared with well to moderately differentiated tumors. This phenomenon is demonstrated in Figure 1G which shows tumor and normal peritumoral mucosa. This finding is consistent with SATB1’s function as a nuclear protein, and the increase of this protein in the nucleus in poorly differentiated CRC likely reflects more pronounced SATB1 activity in chromatin organization as the malignancy of the tumor phenotype increases, consistent with SATB1 acting as a driver of this neoplasm. SATB1 mRNA was found in patient carcinoma samples and in all three of the cell lines we evaluated with RT-PCR. Relatively greater quantities of SATB1 mRNA were found in more poorly differentiated carcinomas, consistent with our immunohistochemistry findings.

Although the specific mechanism by which SATB1 affects the biologic behavior of CRC are unknown, studies of T cell development and breast cancer suggest that SATB1 functions in a global manner via chromatin remodeling and transactivation of the transcription factors of hundreds of gene associated with various signaling pathways, such as the growth factor, Wnt/β-catenin and NF-κB pathways which are known to be crucial in CRC development [7], [16] as well as via DNA repair and genetic mutation which are initiating factors in CRC tumourgenesis [22], [37]. These later three pathways may therefore play a role in the mechanism which couples SATB1 expression to CRC, although this will require further study.

In conclusion, SATB1 was over-expressed in this study in colorectal cancer tissues and cell lines. The expression level of SATB1 showed correlation with tumor differentiation and pTNM stage, and SATB1 was also associated with the expression of various biologic markers (including Cyclin D1, MMP-2, NF-κB, PCNA, APC and BRAF^V600E^). SATB1 promoted growth, colony formation in soft agar and invasion in CRC and reduced neoplastic cell apoptosis. These results all show SATB1 is likely significantly involved in CRC tumor progression and infiltration. Evidence from other studies shows SATB1 over-expression is a common phenomenon in a variety of neoplastic cells, and evidence also suggested SATB1 plays a broad role in the molecular regulation of cancer behavior which thus warrants further investigation.

## Supporting Information

Figure S1 **Colocalization of SATB1 and CEA in CRC cells with confocal microscopy. Bars, 50 µm.**
(PDF)

Figure S2 **Detection of APC loss and BRAF^V600E^ in CRC. A, B:** Via IHC and visualized with AEC, APC protein was detected in well-differentiated adenocarcinoma (**A**) and not in mucinous adenocarcinoma (**B**). Bars, 50 µm. **C:** SSCP analysis of BRAF^V600E^ of 4 CRC cases. Normal bands are indicated with black arrow heads and those of V600E mutation are demonstrated with red arrow heads.
(PDF)

Table S1 **Primary Antibody Details.**
(PDF)
