# Supplementary figures and images for: SATB1 Expression Is Associated with Biologic Behavior in Colorectal Carcinoma In Vitro and In Vivo
Source: PLoS One. 2013 Jan 11;8(1):e47902. doi: 10.1371/journal.pone.0047902 (PMC3543436; doi:10.1371/journal.pone.0047902)

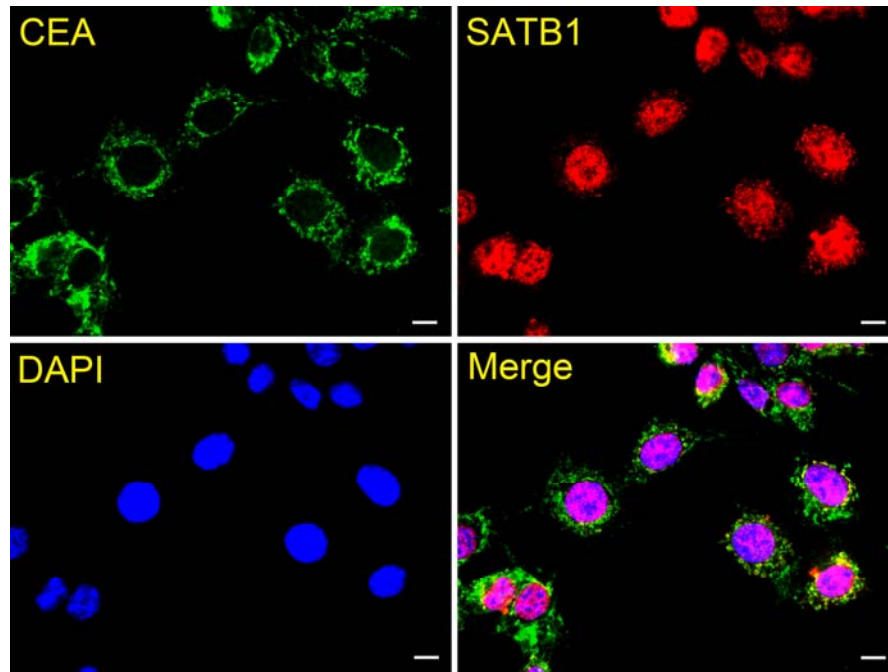

**Figure S1** Colocalization of SATB1 and CEA in CRC cells with confocal microscopy. Bars, 50 $\mu$ m.

Supplement: Figure S1 — Colocalization of SATB1 and CEA in CRC cells with confocal microscopy. Bars, 50 µm. (PDF) [file pone.0047902.s001.pdf]
