# Supplementary material for: SATB1 Expression Is Associated with Biologic Behavior in Colorectal Carcinoma In Vitro and In Vivo
Source: PLoS One. 2013 Jan 11;8(1):e47902. doi: 10.1371/journal.pone.0047902 (PMC3543436; doi:10.1371/journal.pone.0047902)
Supplement: Figure S2 — Detection of APC loss and BRAFV600E in CRC. A, B: Via IHC and visualized with AEC, APC protein was detected in well-differentiated adenocarcinoma (A) and not in mucinous adenocarcinoma (B). Bars, 50 µm. C: SSCP analysis of BRAFV600E of 4 CRC cases. Normal bands are indicated with black arrow heads and those of V600E mutation are demonstrated with red arrow heads. (PDF) [file pone.0047902.s002.pdf]

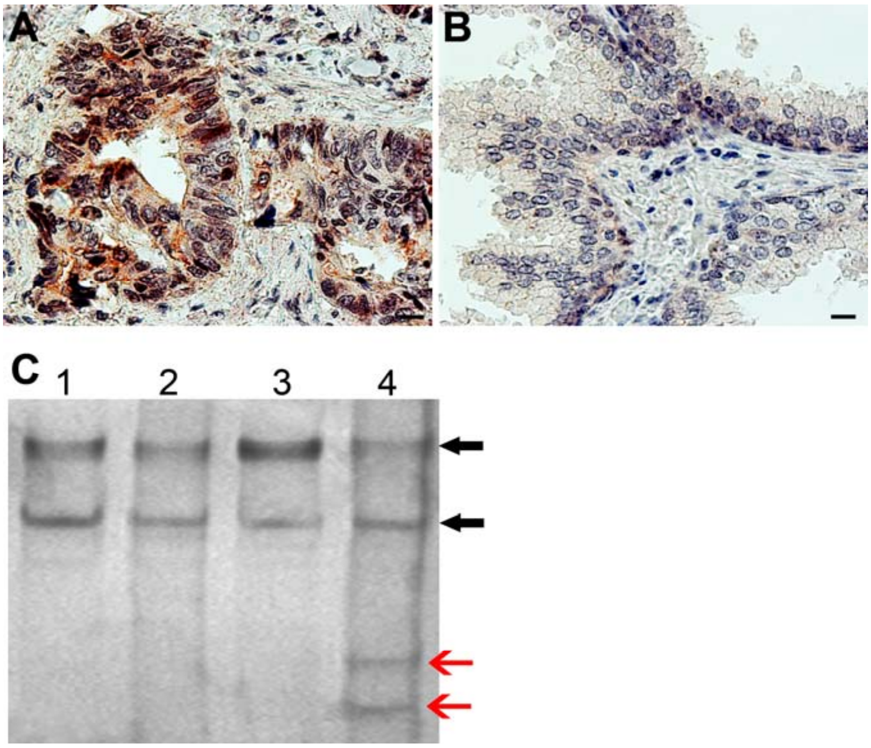

Figure S2 Detection of APC loss and $BRAF^{V600E}$ in CRC.

A, B: Via IHC and visualized with AEC, APC protein was detected in well-differentiated adenocarcinoma (**A**) and not in mucinous adenocarcinoma (**B**). Bars, 50μm.

C: SSCP analysis of $BRAF^{V600E}$of 4 CRC cases. Normal bands are indicated with black arrow heads and those of V600E mutation are demonstrated with red arrow heads.
