# Supplementary material for: SATB1 Expression Is Associated with Biologic Behavior in Colorectal Carcinoma In Vitro and In Vivo
Source: PLoS One. 2013 Jan 11;8(1):e47902. doi: 10.1371/journal.pone.0047902 (PMC3543436; doi:10.1371/journal.pone.0047902)
Supplement: Table S1 — Primary Antibody Details. (PDF) [file pone.0047902.s003.pdf]

Table S1 Primary Antibody Details

Primary antibody	Source	Dilution	Incubation	Supplier
SATB1	Mouse	1:100	4℃, overnight	BD Biosciences, San Jose, CA, USA
P53	Mouse	1:200	4℃, overnight	Zymed Laboratories, San Francisco, CA, USA
PCNA	Mouse	1:2000	4℃, overnight	Cell Signaling, Danvers, MA, USA
Bcl-2	Mouse	1:50	4℃, overnight	Abcam, Cambridge, MA, USA
MMP-2	Mouse	1:50	4℃, overnight	Millipore, Bilerica, MA, USA
NF-κB	Rabbit	1:50	4℃, overnight	Cell Signaling, Danvers, MA, USA
Cyclin D1	Rabbit	1:25	4℃, overnight	Cell Signaling, Danvers, MA, USA
CEA	Rabbit	1:500	4℃, overnight	Zymed Laboratories, San Francisco, CA, USA
APC	Rabbit	1:500	4℃, overnight	Cell Signaling, Danvers, MA, USA
